# Supramolecular Hydrogel Dexamethasone–Diclofenac for the Treatment of Rheumatoid Arthritis

**DOI:** 10.3390/nano14070645

**Published:** 2024-04-08

**Authors:** Yanqin Song, Pufan Yang, Wen Guo, Panpan Lu, Congying Huang, Zhiruo Cai, Xin Jiang, Gangqiang Yang, Yuan Du, Feng Zhao

**Affiliations:** 1 Key Laboratory of Molecular Pharmacology and Drug Evaluation Ministry of Education of China, School of Pharmacy, Yantai University, Yantai 264005, China; songyq89@s.ytu.edu.cn (Y.S.); yangpufan@s.ytu.edu.cn (P.Y.); 18561982002@s.ytu.edu.cn (W.G.); 2914068591@s.ytu.edu.cn (P.L.); huangcy111@s.ytu.edu.cn (C.H.); cccasey01@s.ytu.edu.cn (Z.C.); jacinta113@s.ytu.edu.cn (X.J.); oceanygq@ytu.edu.cn (G.Y.); 2 Yantai Center for Food and Drug Control, Yantai 264005, China

**Keywords:** rheumatoid arthritis, dexamethasone–diclofenac, hydrogel, synergistic anti-inflammatory, NF-κB/COX-2/iNOS pathway

## Abstract

Rheumatoid arthritis (RA) severely affects patients’ quality of life and is commonly treated with glucocorticosteroids injections, like dexamethasone, which may have side effects. This study aimed to create a novel low dose of twin-drug hydrogel containing dexamethasone and diclofenac and explore its potential as a drug delivery system for an enhanced anti-inflammatory effect. Its characterization involved rheology, transmission electron microscope (TEM), Fourier-transform infrared spectroscopy (FTIR), and X-ray diffraction (XRD). Furthermore, the hydrogel demonstrated thixotropic properties. The hydrogel exhibited no cytotoxicity against RAW 264.7 macrophages. Furthermore, the hydrogel demonstrated a significant anti-inflammatory efficacy by effectively downregulating the levels of NO, TNF-α, and IL-6 in lipopolysaccharide (LPS)-activated RAW 264.7 macrophages. The co-delivery approach, when intra-articularly injected in adjuvant-induced arthritis (AIA) rats, significantly alleviated chronic inflammation leading to reduced synovitis, delayed bone erosion onset, and the downregulation of inflammatory cytokines. The biocompatibility and adverse effect evaluation indicated good biological safety. Furthermore, the hydrogel demonstrated efficacy in reducing NF-κB nuclear translocation in LPS-induced RAW 264.7 macrophages and inhibited p-NF-kB, COX-2, and iNOS expression both in RAW 264.7 macrophages and the joints of AIA rats. In conclusion, the findings indicate that the hydrogel possesses potent anti-inflammatory activity, which effectively addresses the limitations associated with free forms. It presents a promising therapeutic strategy for the management of RA.

## 1. Introduction

As a chronic progressive autoimmune inflammatory condition, rheumatoid arthritis (RA) is characterized by a disruption in the regulation of the immune response, instigating a rise in pro-inflammatory cytokines, which is more common in females than males and is often seen in the elderly. The complex pathogenesis involves synovitis and inflammatory cell infiltration, leading to decalcification around the joints, cartilage degradation, and bone erosion [1]. The prevalence of RA differs across various regions, typically falling between 0.5% to 1% of the population [1,2]. As the disease advances, joint defects and the accompanying systemic symptoms contribute to the decline in joint function and disability. This, in turn, negatively impacts the life expectancy of individuals with RA [2]. Presently, nonsteroidal anti-inflammatory drugs (NSAIDs), glucocorticosteroids (GCs), and disease-modifying anti-rheumatic drugs are presented as the main treatment options for RA [3]. Nevertheless, NSAIDs offer only limited effectiveness for short-term inflammation relief [4] Typically, clinical treatment involves the intra-articular injection of GCs, such as dexamethasone. However, the prolonged use of GCs is known to result in severe side effects [5].

The use of intra-articular injectable treatments has garnered increasing attention for enhancing the therapeutic effectiveness of arthritis. This method involves directly injecting drugs into the affected joint, promoting the drug concentration in the target area and minimizing the risk of drug exposure to normal tissues [6]. Nevertheless, in most cases, intra-articular injections typically involve administering the free form of drugs, which can escape quickly from the synovium into the systemic circulation, necessitating frequent injections [7] Currently, supramolecular in situ forming hydrogels have shown promising potential in drug delivery [8]. Supramolecular hydrogels are predominantly based on non-covalent interactions, rendering them generally biocompatible and biodegradable. They stand out as prominent candidates capable of self-delivery of therapeutic agents, due to their molecular simplicity combined with their molecularly engineered self-assembly capabilities [9].

It is worth noting that the extended use of dexamethasone, whether administered intra-articularly or systemically, is associated with significant side effects [10]. Prior research conducted by our group indicated that co-administering GCs with other anti-inflammatory drugs can decrease the dosage of both drugs and mitigate potential side effects associated with individual monotherapies [11]. Furthermore, combining GCs with diclofenac and other NSAIDs offers a synergistic anti-inflammatory action with fewer side effects [12]. Our deduction, therefore, suggests that a combination of both drugs is anticipated to yield synergistic anti-inflammatory activity with reduced toxicity. An ideal approach would involve the development of an injectable hydrogel capable of releasing and sustaining the required drug concentrations in articular joints for prolonged durations. To the best of our knowledge, there have not been any earlier reports of the practical utilization of supramolecular hydrogel for the co-delivery of dexamethasone and diclofenac.

The object of this work was to develop a novel intra-articularly injectable twin-drug hydrogel, combining dexamethasone and diclofenac, with the primary goal of enhancing the therapeutic effect in RA therapy. The initial step involved the preparation of a dexamethasone–diclofenac-loaded hydrogel combination system for intra-articular administration. The supramolecular hydrogel thus fabricated underwent a comprehensive characterization using rheology, transmission electron microscope (TEM), X-ray diffractometer (XRD), and Fourier-transform infra-red (FTIR). An investigation of the in vitro and in vivo anti-inflammatory efficacy and mechanisms of the hydrogel action, using lipopolysaccharide (LPS)-induced RAW 264.7 macrophages and RA rat model, respectively, was aimed at validating its potential as a drug delivery platform for enhancing the therapeutic effect of dexamethasone and diclofenac in RA therapy. Additionally, biocompatibility was assessed through an examination of hemolysis, changes in body weight, hematological parameters, and immune organ index.

## 2. Materials and Methods

### 2.1. Materials

Dexamethasone sodium phosphate (DSP, purity ≥ 99%) and diclofenac sodium (DS, purity ≥ 99%) were purchased from Macklin Biochemical Technology Co., Ltd. (Shanghai, China). Calcium chloride (CaCl_2_, purity ≥ 98%) and Ethylene diamine tetraacetic acid (EDTA, purity ≥ 98%) were purchased from Aladdin Biochemical Technology Co., Ltd. (Shanghai, China). Lipopolysaccharide (LPS) was purchased from Sigma Co., Ltd. (St. Louis, MO, USA). Complete Freund’s Adjuvant (CFA) with 10 mg/mL of heat-killed mycobacterium tuberculosis was supplied by Chondrex Co., Ltd. (Redmond, WA, USA). Biochemical reagents including Cell Counting kit-8 (CCK8), RPMI medium 1640, fetal bovine serum, and 4′,6-diamidino-2-phenylindole (DAPI) were purchased from Beyotime (Shanghai, China). Nitric oxide (NO) kit, ELISA kit for analysis of tumor necrosis factor-a (TNF-a), and interleukin-6 (IL-6) were supplied by KeyGEN BioTECH (Nanjing, China). Alanine transaminase (ALT), aspartate aminotransferase (AST), blood urea nitrogen (BUN), and creatinine (Crea) kits were supplied by Nanjing Jiancheng Co., Ltd. (Nanjing, China). Nuclear factor kappa-B p65 (NF-κB p65), phospho-NF-κB p65 (p-NF-κB p65), cyclooxygenase-2 (COX-2), and inductible nitric oxide synthase (iNOS) antibodies were purchased from Cell Signaling Technology (Danvers, MA, USA).

### 2.2. Cell Culture and Experimental Animals

The School of Pharmacy at Yantai University provided murine RAW 264.7 macrophages. RPMI 1640 medium supplemented with 10% (*v*/*v*) FBS, 100 U/mL penicillin, and 100 µg/m: streptomycin (KeyGEN, Nanjing, China) was used to culture these macrophages. The culture conditions involved maintaining the cells in a humidified environment with 5% CO_2_ in the air at 37 °C. When macrophages reached 80% confluence, they were trypsinized using a 0.25% trypsin/EDTA solution in PBS.

Pengyue Animal Co., Ltd. (Qualification No. SCXK 20220006, Jinan, China) supplied male Sprague–Dawley rats (each weighing 180–220 g) which were kept in a 12/12 h light/dark schedule at 22 °C, and were given unlimited access to food and water. All animal studies were authorized by Yantai University’s Animal Ethics Committee (No. 20220908-11) and followed the National Institutes of Health’s Guide for the Care and Use of Laboratory Animals.

### 2.3. Fabrication of Supramolecular Hydrogel

DSP and DS were solubilized in distilled water to obtain a DSP–DS aqueous solution. A CaCl_2_ solution was made by dissolving the appropriate amount of CaCl_2_ in DI water. Subsequently, solutions of DSP–DS and CaCl_2_ with varying weight ratios were combined at room temperature and allowed to stir, resulting in the formation of DSP–DS supramolecular hydrogel overnight.

### 2.4. TEM Analysis

The DSP–DS supramolecular hydrogel was deposited on the grid. The grid was rinsed with DI water and stained with phosphotungstic acid (0.5 wt%) for 3 min prior to TEM analysis (Model Talos F200X G2, Thermo Scientific, Waltham, MA, USA). The acceleration voltage is 200 KV.

### 2.5. Rheological Analysis

A Haake Mars40 rheometer (Thermo Scientific, Karlsruhe, Germany) equipped with a 40 mm cone-plate was employed to conduct rheological tests for the DSP–DS supramolecular hydrogel (0.5 mL). With a strain amount of 1%, a frequency sweep ranging from 0.1 to 100 rad/s was carried out at 25 °C. A strain sweep (1 Hz, 25 °C) and a strain amplitude sweep (1 Hz, 25 °C) were performed, covering low-magnitude strain (c = 1%) and high-magnitude strain (c = 100%).

### 2.6. FTIR Analysis

FTIR analysis of samples, including free DSP+DS powder and DSP–DS xerogel, was carried out using an IR spectrometer (Model Nicolet iS20, Thermo Scientific, USA). The analysis covered a spectral range from 4000 cm^−1^ to 500 cm^−1^.

### 2.7. XRD Analysis

An automatic X-ray diffractometer (Ultima IV, Rigaku Corporation, Tokyo, Japan) was employed to record the XRD patterns of samples employing CuKa radiation in the 2θ range of 10–70°.

### 2.8. In Vitro Drug Release

A dialysis bag (MW 3500 Da) was sealed with the donor compartments of vertically modified diffusion cells, and 0.5 mL of DSP–DS supramolecular hydrogel (0.25 wt%) was added. PBS solution (10 mL) was added into the release compartment as the release media and was left to incubate at 37 °C for in vitro drug release. At specified intervals, the PBS solution was proceeded for HPLC analysis of released DSP and DS. Fresh PBS solution replaced the release medium in each collection.

### 2.9. Hemolytic Assay

Hemocompatibility assessments of DSP–DS supramolecular hydrogel, free DSP, and free DSP+DS were conducted using a spectrophotometric method. Fresh blood was drawn from the rabbit’s heart, and RBCs were isolated through a 15 min centrifugation at 2500 rpm. Following thorough washing, solutions of DSP–DS supramolecular hydrogel, free DSP, and free DS at different concentrations were added to 2% (*v*/*v*) RBCs for further evaluation.

Following mixing with a vortex, the RBC suspensions were subjected to incubation in a thermostatic water bath at 37 °C for 4 h. The negative and positive controls were 1.0 × 10^4^ mg/mL solutions of NaCl and 0.1% SDS, respectively. Centrifugation of the RBC suspensions was carried out for 10 min at 3000 rpm, and supernatant from individual samples (100 μL) was added to a 96-well plate. SpectraMax i3x microplate reader (Molecular Devices Co., Ltd., San Jose, CA, USA) was used to detect the free hemoglobin released from RBCs at 540 nm.

### 2.10. Cytotoxicity Study

The in vitro cytotoxicity of the DSP–DS hydrogel and DSP+DS aqueous solution against RAW 264.7 cell was measured using CCK-8 assay. Using a density of 1 × 10^4^ cells/well, cells were seeded into 96-well plates and left for overnight culture at 37 °C in a CO_2_ incubator. The DSP–DS hydrogel or the free DSP+DS aqueous solution at drug concentrations ranging from 2.5–20 μM (equal doses of DSP and DS) was added. Following an incubation of 24 h, CCK-8 solution (20 μL) was added. The incubation was continued for another 2 h. A microplate reader (SpectraMax i3x, San Diego, CA, USA) was then utilized to record the optical absorbance at 450 nm. The control comprised untreated cells. All experiments were carried out thrice and the cell viability was computed using the expression given below:

% cell viability = absorbance of test specimen/absorbance of control

### 2.11. In Vitro Anti-Inflammatory Activities

LPS-activated RAW 264.7 cells were employed to determine the in vitro anti-inflammatory efficacy of the DSP–DS supramolecular hydrogel. A 24-well plate was employed to seed the cells at a density of 1.0 × 10^5^ cells/well followed by overnight incubation in a 5% CO_2_ incubator at 37° C. After a passage of 24 h, the cells underwent pre-treatment with free DSP+DS or DSP–DS hydrogel (DSP and DS were dosed at 5 μM and 4 μM, respectively) for 2 h. LPS (1 μg/mL) was employed to stimulate the cells for an additional 24 h. Collection of the cell culture supernatants was followed by the measurement of the levels of NO, TNF-α, and IL-6. Untreated cells served as the control.

### 2.12. Immunofluorescence Staining

Cells were seeded at a concentration of 1 × 10^5^ cells per well over a sterile glass coverslip in 6-well plates and allowed to culture in RPMI with 2% FBS overnight. Following 24 h, the cells underwent pre-treatment with free DSP (10 μM), free DSP+DS solution, or DSP–DS hydrogel (each dose of DSP and DS was 5 μM and 4 μM, respectively) for 2 h. Subsequently, LPS induction (1 μg/mL) was carried out for 12 h, followed by rinsing with PBS, fixation with 4% paraformaldehyde for 20 min, and treatment with 0.1% Triton X-100 for 20 min. Overnight incubation of the cells with anti-NF-κB p65 primary antibody at 4 °C was subsequently carried out, which was followed by treatment with Alexa Fluor 594 secondary antibody for 1 h in the dark. DAPI and anti-fading buffer were ultimately added, and a confocal laser scanning microscope (LSM800, Zeiss, Oberkochen, Germany) captured the protein fluorescence signal images.

### 2.13. Adjuvant-Induced Arthritis Model and Monitoring

Adjuvant-induced arthritis (AIA) model, a widely recognized arthritis disease model, was established as the right hind paw of SD rats was subcutaneously injected with a complete Freund’s adjuvant (CFA, 100 μL) containing heat-killed mycobacteria (10 mg/mL) [13]. The development of paw swelling and joint inflammation in rats was regularly monitored, and appropriate care, as per our previous study, was administered to ensure ad libitum access to food and water throughout the progression of the disease [14].

After 12 days of the arthritis induction, the rats were divided randomly into five groups (n = 8): (i) control group (CON), (ii) AIA group (CFA-challenged and injected with sterile PBS, AIA), (iii) free-DSP-treated group challenged with CFA (free DSP, 1 mg/kg), (iv) free DSP and DS challenged with CFA (free DSP+DS, 0.5 mg/kg DSP + 0.4 mg/kg DS), and (v) DSP+DS-loaded-hydrogel-treated group challenged with CFA (DSP–DS hydrogel, 0.5 mg/kg DSP + 0.4 mg/kg DS). The treatments were started from day 12 by intra-articularly injecting 50 µL of the test substances directly into the knee joint area of arthritic rats every day (iii and iv), or three days (v) after CFA immunization.

### 2.14. Assessment of Arthritis Progression upon CFA Administration

For each rat, the volume of the right paw was recorded before CFA injection by utilizing a water plethysmometer and, subsequently, at days 3–27. This is referred to as primary swelling. The body weight was simultaneously recorded. From day 11 onwards, the volume of the left paw was also measured (as secondary swelling) every two days until the conclusion of the experiment.

Three independent observers assessed the severity of arthritis. Following the second induction, periodic observation of the severity of joint inflammation in the rats was carried out, until they were finally sacrificed. The arthritis index was evaluated using a scale ranging from 0 to 4, employing the following criteria: 0 indicated no edema, 1 denoted minor edema and limited erythema, 2 represented minor edema and erythema beginning at ankle and extending up to tarsal bone, 3 indicated moderate edema and erythema covering the area from the ankle to tarsal bone, and 4 signified edema and erythema beginning at ankle and covering the entire leg. By examining the severity observed in all four paws, the arthritis score for individual rats was determined by the method of summation, the maximum score being 16. Additionally, the number of swollen joints was recorded and subjected to analysis.

### 2.15. Radiography and Histological Analysis

On day 28 of the experiment, the rats were anesthetized and positioned on a radiographic box, maintaining a 90 cm distance from the source of radiation. An X-ray machine with a 40 kW exposition was employed to conduct the radiographic analysis of arthritic hind paws, keeping the X-ray exposure time to 15 s.

H&E staining of the ankle joints, heart, lung, kidney, liver, and spleen was subsequently carried out. A light microscope (Model IX71 Olympus, Tokyo, Japan) was used for making observations.

### 2.16. Biochemical Analysis

On day 28 following immunization, rats were sacrificed. The blood from each group was collected, followed by the measurement of serum and cellular levels of NO, TNF-α, and IL-6 levels, in accordance with the protocol outlined by the manufacturer. The levels of the ALT, AST, BUN, and Crea in serum were also analyzed.

### 2.17. Index of Spleen and Thymus Assay

On day 28, the rats were euthanized under anesthesia, followed by immediate excision and weighing of the spleen and thymus. The spleen and thymus indices were calculated as the ratio (mg/g) of the wet weight of the corresponding organ to the body weight.

### 2.18. Western Blotting

Lysates from RAW 264.7 cells or AIA rat joints were prepared using ice-cold RIPA buffer. The protein concentration was determined by employing a BCA kit (Beyotime, Shanghai, China). Equal quantities of proteins (30 μg) were separated on 10% SDS-PAGE, transferred onto a polyvinylidene fluoride membrane (0.22 μm, Millipore Co., Bedford, MA, USA), and 5% fat-free dry milk in Tris-buffered saline with Tween-20 (TBST) was used to block them for 1 h at room temperature. The membranes were incubated overnight at 4 °C with primary antibodies against anti-p-NF-κB, anti-NF-κB, anti-COX-2, and anti-iNOS, with anti-β-actin functioning as an internal reference. The membranes were incubated with an HRP-conjugated secondary antibody after three washes with TBST, and chemiluminescence was visualized using ImageQuant LAS4000 (GE Healthcare, Wayne, PA, USA). Image-Pro Plus 6.0 (Media Cybermetics, MD, USA) was used to carry out the semi-quantitative analysis, and individual experiments were replicated thrice.

### 2.19. Statistical Analysis

The results, presented as mean ± S.D, were analyzed using GraphPad Prism 8.0 software. Statistical significance was assessed through Student’s *t*-test for two-group comparisons and one-way ANOVA for multiple groups, with a significance level set at *p* < 0.05.

## 3. Results and Discussion

### 3.1. Formation of DSP–DS Supramolecular Hydrogel

**Figure 1:**
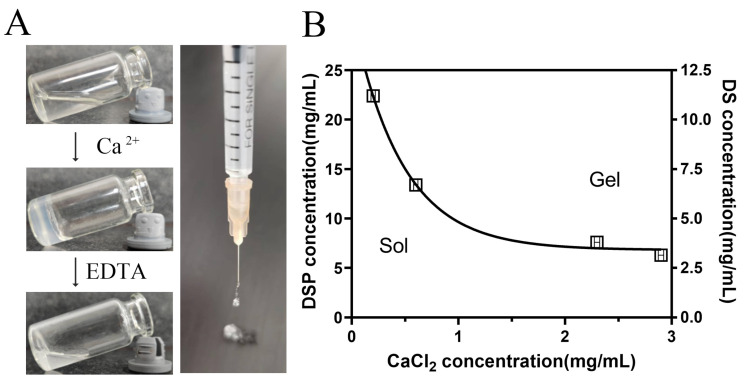
The formation of DSP–DS hydrogel. (**A**) Photographs showing DSP+DS solution (without Ca^2+^), hydrogel (with Ca^2+^), and EDTA-induced sol–gel–sol transition and injectable of hydrogel. (**B**) Phase diagram of the sol–gel transition as function of DSP, DS, and CaCl_2_ concentrations.

In the treatment of RA, addressing non-targeted systemic cytotoxicity and the limited bioavailability of drug therapies is a key priority. Developing drug delivery systems for biomedical purposes is a major focus. Many research articles have concentrated on drug delivery systems for combined diclofenac and dexamethasone, utilizing liposomes and polylactide nanoparticles. These systems are often created using elaborate synthetic reactions or formulations. According to several literature reports, several steroid drugs, including betamethasone phosphate, hydrocortisone phosphate, and dexamethasone phosphate, characterized by a rigid hydrophobic core featuring four fused rings, multiple hydroxyl moieties, and a phosphate group, have the capability to self-assemble into nanofiber hydrogels through diverse molecular interactions [15]. In this study, an injectable nanofiber hydrogel with shear-thinning characteristics was fabricated by combining DSP with DS, which was easy to prepare and apply. This allows for the sustained release of both DSP and DS, enhancing the synergistic therapy for RA. After incubating the hydrogel with an EDTA aqueous solution, a potent Ca^2+^ chelator, it transforms into a transparent solution (Figure 1A).

The hydrogelation response of DSP and DS to varying concentrations of Ca^2+^ was investigated. As depicted in Figure 1B, the sol–gel transition was predominantly influenced by CaCl_2_ and DSP concentrations. Higher concentrations led to rapid gelation, indicating increased cross-linking density. Interestingly, the DS concentration had minimal impact on gel formation. This hydrogel, distinct from prior studies, demonstrates the capability to encapsulate other drugs. Formed via non-covalent interactions, for instance, hydrogen bonding and hydrophobic interactions among steroid moieties, the nanofiber hydrogel also involves the co-ordination between phosphate groups and calcium ions. This unique structure enables direct injection into tissues with minimal invasiveness.

### 3.2. Characterization of DSP–DS Hydrogel

**Figure 2:**
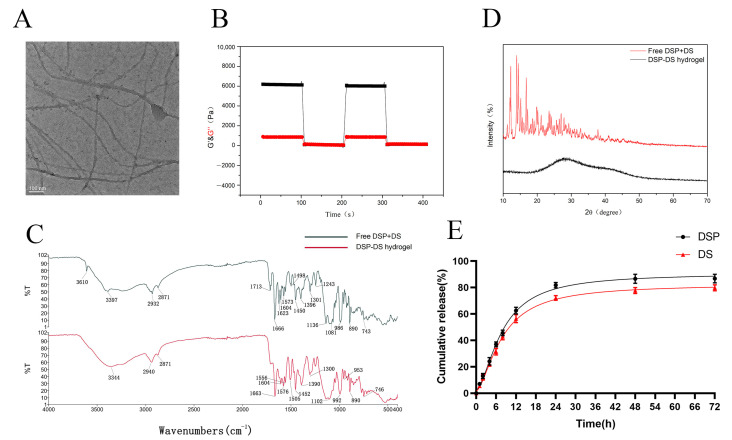
The characterization of DSP–DS hydrogel. (**A**) Representative TEM image of DSP–DS hydrogel. (**B**) Rheological behavior of DSP–DS hydrogel: G′, storage modulus; G″, loss modulus. (**C**) FTIR spectra of DSP+DS powder and DSP–DS hydrogel. (**D**) XRD patterns of DSP+DS powder and DSP–DS hydrogel. (**E**) Cumulative release profiles of DSP and DS from DSP–DS hydrogel in PBS of pH 7.4 at 37 °C.

TEM was utilized to analyze the microscopic structural details of the DSP–DS supramolecular hydrogel. As depicted in Figure 2A, an extended nanofiber network was observed. Nanofibers having diameters of approximately 15 nm and lengths spanning a few micrometers predominantly constituted the hydrogel. The flexible and uniform nanofibers intertwined, creating a stable three-dimensional supramolecular hydrogel. Additionally, the confirmation of the DSP–DS hydrogel formation was achieved through rheological behavior testing (Figure 2B). The storage modulus (G′) was notably larger than the loss modulus (G″) was observed, indicating the presence of a representative elastic network within this hydrogel. This characteristic is commonly shared by all hydrogels with either non-covalent or covalent crosslinking.

Subsequently, the DSP–DS supramolecular hydrogel was subjected to XRD and FTIR analysis. The FTIR result (Figure 2C) showed the symmetric and anti-symmetric stretches of phosphate gave rise to bands at 986 and 1081 cm^−1^ which are evident in the FTIR spectra of free DSP+DS. These bands transitioned to higher wavenumbers (992 and 1102 cm^−1^) in the DSP–DS hydrogel, suggesting the involvement of the phosphate in co-ordination bonds [14]. Meanwhile, the characteristic peaks of the DS in free DSP+DS (743 and 1573 cm^−1^) is consistent with that in the DSP+DS hydrogel (746 and 1576 cm^−1^), suggesting the minimal effect in co-ordination bonds [16,17]. The XRD pattern (Figure 2D) showed the crystalline properties of the physical mixture of DS powder and DSP were clearly evident, revealing specific reflections within the 10–70° range. Notably, the distinct reflections associated with DSP and DS powder were no longer present in the DSP+DS hydrogel. The results at 28.5° and 40.7° show two typical wide peaks of the DSP+DS hydrogel, rather than high-strength crystalline peaks, which are caused by the random arrangement of the constituent molecules and may be attributed to the amorphous state of the DSP-Ca^2+^ complex [18]. XRD studies show that the free DSP+DS is loaded in an amorphous form and well-encapsulated in the hydrogel.

Diverging from the traditional method of physically encapsulating drugs, the design and synthesis of supramolecular hydrogels provide us with the unique capability to incorporate therapeutic agents directly during the process of hydrogelation. This approach not only grants control over the drug payload, it additionally serves as a matrix facilitating the controlled release of the encapsulated drugs [19]. The release patterns of DSP and DS were examined in PBS at 37 °C. As shown in Figure 2E, a two-stage release, comprising an initial rapid release of DSP and DS within the first 20 h, was followed by a relatively accumulative release over the next 72 h.

### 3.3. Effect of DSP–DS Hydrogel on the Cytotoxicity and Level of NO, TNF-α, and IL-6 in RAW264.7

**Figure 3:**
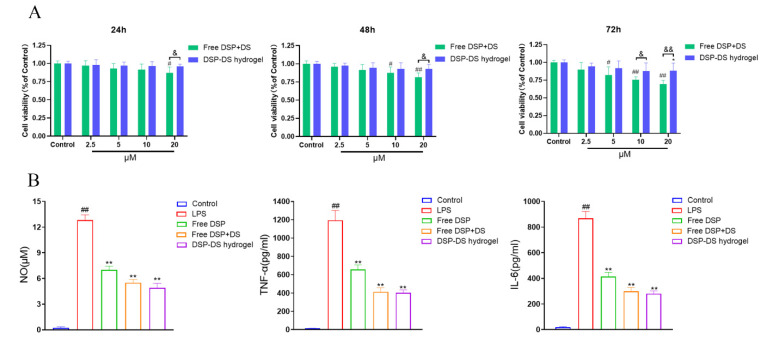
Effect of DSP–DS hydrogel on the cytotoxicity and level of NO, TNF-α, and IL-6 in RAW264.7. (**A**) The viability of RAW264.7 macrophages after 24, 48, and 72 h exposure to free DSP+DS solution and DSP–DS hydrogel at various concentrations, determined by CCK-8 assay (n = 5). (**B**) The levels of NO, TNF-α, and IL-6 of LPS-activated RAW264.7 macrophages after 24 h exposure to free DSP solution, free DSP+DS solution, and DSP–DS hydrogel. Data are presented as mean ± S.D. ^#^ *p* < 0.05, ^##^ *p* < 0.01 vs. the control group; * *p* < 0.05, ** *p* < 0.01 vs. the LPS group; ^&^ *p* < 0.05, ^&&^ *p* < 0.01.

Ensuring the non-toxic character of the formulation is a critical consideration before proceeding with further in vivo applications [20]. The CCK-8 assay was employed to evaluate the cell viability of the DSP–DS hydrogel compared to free DSP+DS in RAW264.7 macrophages. As illustrated in Figure 3A, the DSP–DS hydrogel displayed no discernible cytotoxicity towards RAW264.7 macrophages following even 24 h of incubation, and at dual-drug concentrations as high as 20 μM. In contrast, free DSP+DS significantly reduced cell viability as the concentration of the drug increased to 20 μM, with a more pronounced decrease observed after 48 and 72 h of incubation. Despite a slight decline in cell viability induced by the DSP–DS hydrogel after 48 and 72 h of incubation, the cell survival rate remained above 75%. These findings strongly suggest that the proposed DSP–DS hydrogel is non-toxic and holds promise as an anti-inflammatory drug delivery system.

In several inflammatory diseases, including RA, activated macrophages secrete inflammatory mediators including NO, TNF-α, and IL-6 [21]. Synovial macrophages are identified as a primary source of TNF-α in RA [22]. While TNF-α is a key player in our immune response, its overproduction can kickstart the inflammatory process, leading to the pathogenesis of RA [23]. In various physiological and pathological conditions, NO plays a key role as a proinflammatory factor [24]. IL-6, often induced by TNF-α, is a common inflammatory cytokine that contributes to RA inflammation by further activating lymphocytes and macrophages [25]. Assessing the downregulation of NO, IL-6, and TNF-α in LPS-induced RAW264.7 cells is a preferred method for evaluating the anti-inflammatory effects of drugs [26]. Therefore, suppressing the production of these inflammatory mediators emerges as a crucial target for treating RA [27]. As shown in Figure 3B, RAW 264.7 macrophages, when stimulated with LPS, exhibited a significant rise in NO, TNF-α, and IL-6 secretion in the culture supernatant. Treatment with free DSP, free DSP+DS, and DSP–DS hydrogel markedly attenuated the release of NO, TNF-α, and IL-6 compared to LPS alone. Importantly, the anti-inflammatory efficacy of DSP–DS hydrogel closely resembled that of free DSP+DS, suggesting that the hydrogel’s action with DSP and DS doses does not compromise its anti-inflammatory activity. The proposed DSP–DS supramolecular hydrogel thus holds promise as a drug delivery system for RA treatment.

### 3.4. Effect of DSP–DS Hydrogel on the NF-κB/COX-2/iNOS Pathway Suppression in LPS-Activated RAW264.7

The NF-κB pathway is a crucial pathway regulating inflammation [28]. NF-κB is present in the cytoplasm in its inactive form [29]. Upon activation by LPS, NF-κB translocates to the nucleus and stimulates the transcription of genes [30]. NF-κB has an indispensable function in regulating the expression of inflammation-related enzymes, iNOS and COX-2, to be precise [31]. Binding sites for NF-κB have been found in the promoter regions of the COX-2 and iNOS genes. These genes are involved in producing inflammatory mediators like IL-6, TNF-α, and NO [32]. Hence, it is essential to modulate the NF-κB/COX-2/iNOS signaling pathway to regulate inflammation [33].

**Figure 4:**
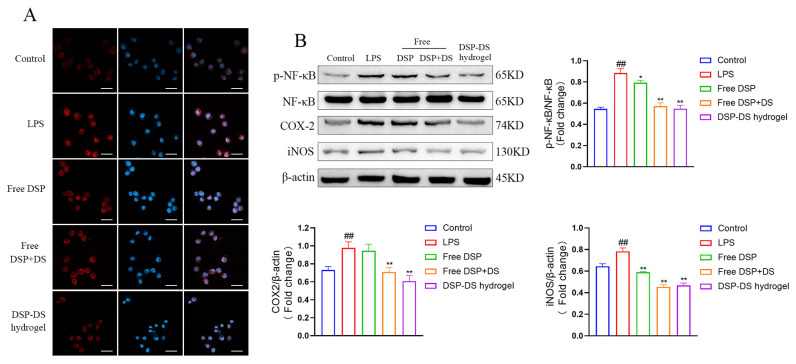
Effect of DSP–DS hydrogel on the NF-κB/COX-2/iNOS pathway suppression in LPS-activated RAW264.7. (**A**) The translocation of NF-κB p65 of LPS-activated RAW264.7 macrophages after 12 h exposure to free DSP solution, free DSP+DS solution, and DSP–DS hydrogel were detected by immunofluorescence staining (the scale bar = 50 μm). (**B**) The expression of p-NF-κB, NF-κB, COX-2, and iNOS of RAW264.7 macrophages was detected by western blot (n = 3). Data are presented as mean ± S.D. ^##^ *p* < 0.01 vs. the control group; * *p* < 0.05, ** *p* < 0.01 vs. the LPS group.

Thus, NF-κB transduction is a crucial process in the development of inflammation [34]. In Figure 4A, immunofluorescence was utilized to observe the NF-κB p65 distribution in RAW264.7 macrophages. In the control cells, NF-κB p65 (red fluorescence) was predominantly localized in the cytoplasm around blue fluorescent DAPI-stained nuclei. The red and blue fluorescence merged following 12 h of LPS stimulation, thereby suggesting the rapid translocation of NF-κB p65 to the nucleus. Treatment with free DSP or free DSP+DS reduced NF-κB p65 translocation in comparison to LPS alone, and the DSP–DS hydrogel group exhibited a more pronounced effect. Protein level alterations were observed through western blotting for p-NF-κB, NF-κB, COX-2, and iNOS proteins. This aimed to explore potential pathways contributing to the observed anti-inflammatory effects. The DSP–DS hydrogel demonstrated a significant inhibition of the NF-κB pathway activation, concomitantly suppressing the expression of iNOS and COX-2, as illustrated in Figure 4B.

### 3.5. Effect of DSP–DS Hydrogel on the Therapeutic Efficacy in AIA Rats

**Figure 5:**
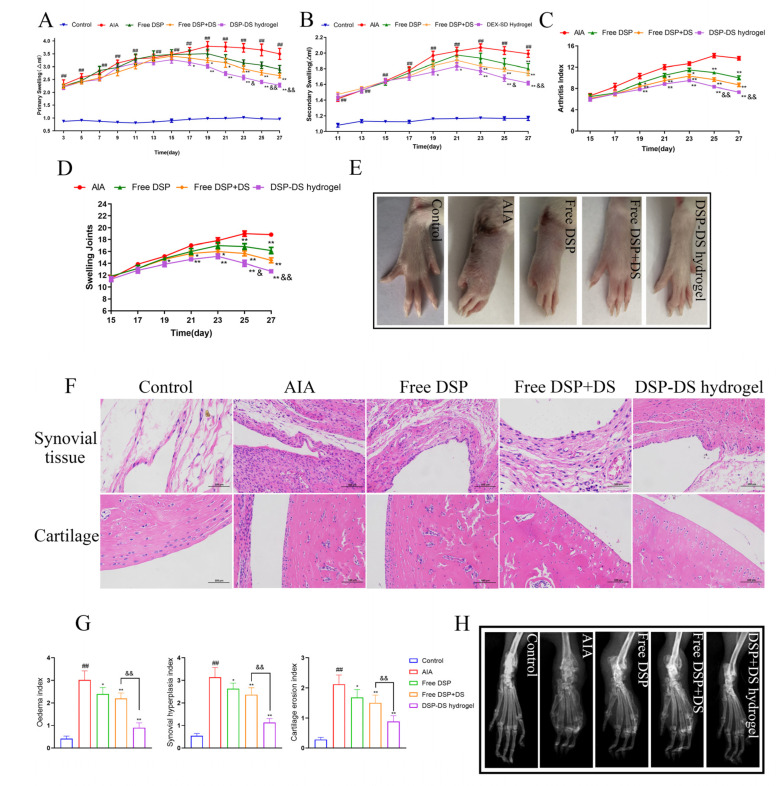
Effect of DSP–DS hydrogel on the therapeutic efficacy in AIA rats. (**A**) Primary swelling. (**B**) Secondary swelling. (**C**) Arthritis index. (**D**) Swelling joints. (**E**) Representative photographs of hind paws. (**F**) Histological analysis of synovial tissue and cartilage in ankle joints (H&E staining, 200× magnification). (**G**) Histopathological indices for edema, synovial hyperplasia, and cartilage erosion. (**H**) Radiographic changes. Data are presented as mean ± S.D. ^##^ *p* < 0.01 vs. the control group; * *p* < 0.05, ** *p* < 0.01 vs. the AIA group; ^&^ *p* < 0.05, ^&&^ *p* < 0.01 vs. the free DSP+DS group.

To assess the anti-inflammatory effects, AIA rats were treated with the DSP–DS hydrogel through intra-articular delivery. Subsequently, hind paw photographs, primary and secondary swelling measurements, the arthritis index, and the count of swelling joints were recorded for 28 days post the initial immunization. Primary and secondary swelling of the paw, serving as indicators of arthritis, were measured to assess the anti-inflammatory action. Figure 5A,B illustrates a substantial increase in paw swelling in the AIA group, confirming the establishment of the AIA rat model. After the intra-articular administration of free DSP, free DSP+DS, and DSP–DS hydrogel, the paw-swelling time curves in arthritic rats demonstrated a notable decrease. It is important to note that the DSP–DS hydrogel group demonstrated the highest therapeutic efficacy. This outcome is attributed to the enhanced long circulation time and accumulation capability of the drug at inflamed joints. The arthritis index (Figure 5C) and swelling of joints (Figure 5D) consistently demonstrated suppression, aligning with the paw-swelling results. Moreover, macroscopic observations of the hind leg morphology supported these findings (Figure 5E).

A histopathological examination of the ankle joints in AIA rats revealed severe inflammation and cartilage damage. The group treated with DSP–DS hydrogel manifested a significant reduction in cartilage erosion and synovial inflammation, showing a clear interface and minimal inflammatory cell infiltration (Figure 5F). A quantitative analysis validated the effective alleviation of arthritic symptoms in AIA rats treated with DSP–DS hydrogel, with approximately 1.5-fold lower synovial inflammation and cartilage destruction compared to free DSP+DS (Figure 5G). An X-ray assessment demonstrated reduced bone erosion in AIA rats treated with DSP–DS hydrogel (Figure 5H). Compared to the rats treated with hydrogel, the DSP–DS hydrogel group exhibited enhanced protection against inflammation-induced damage to bone tissue. The DSP–DS hydrogel group displayed improved bone structure and reduced erosion, indicating enhanced drug accumulation in the inflamed joints, thereby improving the anti-inflammatory effects and alleviating the paw-swelling symptoms.

### 3.6. Effect of DSP–DS Hydrogel on the Level of NO, TNF-α and IL-6 in Serum of AIA Rats

**Figure 6:**
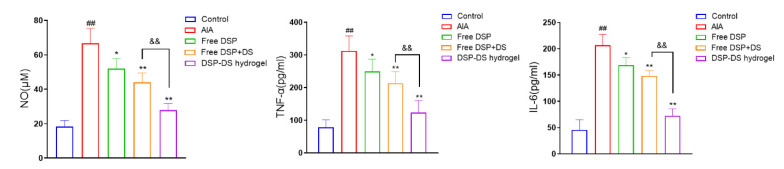
Effect of DSP–DS hydrogel on the level of NO, TNF-α, and IL-6 in serum of AIA rats. The levels of NO, TNF-α, and IL-6 in the serum of AIA rats were examined. Data are presented as mean ± S.D. ^##^ *p* < 0.01 vs. the control group; * *p* < 0.05, ** *p* < 0.01 vs. the AIA group; ^&&^ *p* < 0.01.

The pro-inflammatory cytokines NO, IL-6, and TNF-α have a vital function in the onset and development of RA [35]. As shown in Figure 6, the levels of inflammatory cytokines, including serum NO, TNF-α, and IL-6, were moderately reduced in the free DSP or free DSP+DS group compared to the AIA group. Notably, the DSP–DS hydrogel group exhibited even lower levels of these inflammatory markers compared to both the free DSP and free DSP+DS groups.

### 3.7. Effect of DSP–DS Hydrogel on the NF-κB/COX-2/iNOS Pathway Suppression in AIA Rats

**Figure 7:**
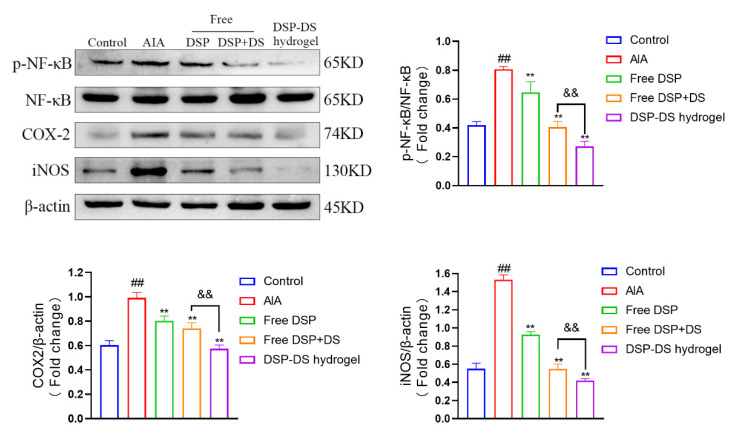
Effect of DSP–DS hydrogel on the NF-κB/COX-2/iNOS pathway suppression in AIA rats. The expression of p-NF-κB, NF-κB, COX-2, and iNOS in joints of AIA rats was detected by western blot (n = 3). Data are presented as mean ± S.D. ^##^ *p* < 0.01 vs. the control group; ** *p* < 0.01 vs. the AIA group; ^&&^ *p* < 0.01.

As anticipated (Figure 7), the AIA rat group exhibited a higher ratio of p-NF-κB/NF-κB and an increased expression of COX-2 and iNOS. Notably, these effects were significantly inhibited by free DSP or free DSP+DS treatments, with the DSP–DS hydrogel group showing the lowest values for the ratio of p-NF-κB/NF-κB and the expression of COX-2 and iNOS.

### 3.8. Evaluation of Biocompatibility and Adverse Effects on DSP–DS Hydrogel

**Figure 8:**
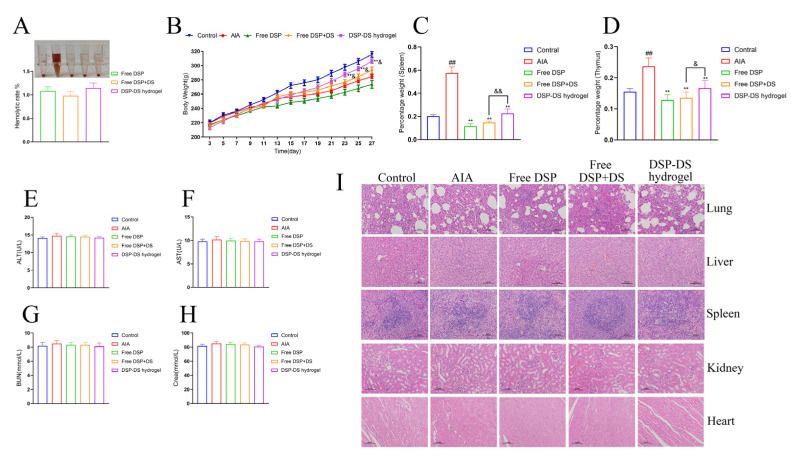
Evaluation of biocompatibility and adverse effects on DSP–DS hydrogel. (**A**) Hemocompatibility assay. (**B**) Body weight of rats was recorded from day 3 to 27. (**C**) Immune organ index of spleen. (**D**) Immune organ index of thymus. The levels of ALT (**E**), AST (**F**), BUN (**G**), and Crea (**H**) in serum of rats were measured. (**I**) Histological analysis of lung, liver, spleen, kidney, and heart in rats (H&E staining, 200× magnification). Data are presented as mean ± S.D. ^##^ *p* < 0.01 vs. the control group; * *p* < 0.05, ** *p* < 0.01 vs. the AIA group; ^&^ *p* < 0.05, ^&&^ *p* < 0.01 vs. the free DSP+DS group.

The hemolytic assay was executed to assess the safety attributes of the DSP–DS hydrogel. As depicted in Figure 8A, saline was taken as the negative control, and 0.1% SDS functioned as the positive control. The DSP–DS hydrogel exhibited no significant hemolysis (<3%).

The long-term administration of frequent and high doses of GCs in autoimmune disorders, as part of GC therapy, typically leads to undesirable systemic side effects such as immunosuppression and abnormal changes in body weight [36]. In the duration of the entire treatment procedure, rats in the hydrogel treatment group did not display symptoms of anxiety, anorexia, or any other abnormalities. Although all treated groups experienced a substantial decrease in weight gain in comparison to the control group, signifying the substantial impact of AIA inflammation, the encapsulation of DSP and DS in DSP–DS hydrogel notably mitigated the unwanted effect of weight loss (Figure 8B). This implies that our treatment regimen does not induce remarkable weight loss. The AIA rat model, which mimics immune dysfunction caused by autoimmune diseases, typically uses immune organ indices to assess the overall functioning of the immune system in the body. The results showed that free DSP or free DSP+DS significantly reduced the splenic and thymic indices in AIA rats, indicating immunotoxicity caused by free DSP. In contrast, the DSP–DS hydrogel notably improved thymus and spleen atrophy in AIA rats, suggesting a potential for restoring immune organ function with good biocompatibility and a potent anti-RA effect (Figure 8C,D). Various parameters, especially the liver (ALT, AST) and kidney (BUN, Crea), were also assessed (Figure 8E,H). All the indicators were in the normal range, suggesting no significant damage to these organs. Further examination through H&E staining did not show any noticeable abnormalities or lesions in the major organs (lung, liver, spleen, kidney, and heart), confirming the absence of any tissue injury (Figure 8I).

## 4. Conclusions

In this study, a successful dual-drug hydrogel formulation of DSP and DS was prepared and verified for the treatment of RA. Both drugs, DSP and DS, are commonly used to treat RA and inflammatory conditions. However, their therapeutic efficacy and side effects were found to be unsatisfactory. The hydrogel formulation exhibited desirable features such as mechanical strength, prolonged release, injectability, and biosafety. The treatment with DSP–DS hydrogel was found to significantly inhibit the inflammatory response, effectively reduce chronic inflammation, and delay bone erosion in RA rats. The formulation has three main advantages: it has a high therapeutic potential with a cost-effective and simple synthesis approach, it reduces drug doses and related side effects through the combination of DSP and DS, and it improves the RA condition in inflamed joints by co-delivering both drugs from the hydrogel.

To conclude, the findings indicate that the use of DSP–DS hydrogel via intra-articular injection can expedite anti-RA therapy procedures, leading to enhanced therapeutic outcomes while minimizing dose-related side effects associated with the unconstrained forms of the medications. The anti-inflammatory process potentially occurs by impeding the NF-κB/COX-2/iNOS pathway. Overall, this investigation highlights the substantial potential of DSP–DS hydrogel as a viable approach for treating RA and other inflammatory conditions. It is important to highlight that the supramolecular hydrogel platform used in this study can be conveniently functionalized and prove to be therapeutically effective, making it highly advantageous for in vivo applications, especially for the co-delivery of anti-RA medications to achieve a synergistic effect.

## Data Availability

The data are available upon request from the authors.
